# Graded, Dynamically Routable Information Processing with Synfire-Gated Synfire Chains

**DOI:** 10.1371/journal.pcbi.1004979

**Published:** 2016-06-16

**Authors:** Zhuo Wang, Andrew T. Sornborger, Louis Tao

**Affiliations:** 1 Center for Bioinformatics, National Laboratory of Protein Engineering and Plant Genetic Engineering, College of Life Sciences, Peking University, Beijing, People’s Republic of China; 2 Department of Mathematics, University of California, Davis, Davis, California, United States of America; 3 Center for Quantitative Biology, Peking University, Beijing, People’s Republic of China; Université Paris Descartes, Centre National de la Recherche Scientifique, FRANCE

## Abstract

Coherent neural spiking and local field potentials are believed to be signatures of the binding and transfer of information in the brain. Coherent activity has now been measured experimentally in many regions of mammalian cortex. Recently experimental evidence has been presented suggesting that neural information is encoded and transferred in packets, i.e., in stereotypical, correlated spiking patterns of neural activity. Due to their relevance to coherent spiking, synfire chains are one of the main theoretical constructs that have been appealed to in order to describe coherent spiking and information transfer phenomena. However, for some time, it has been known that synchronous activity in feedforward networks asymptotically either approaches an attractor with fixed waveform and amplitude, or fails to propagate. This has limited the classical synfire chain’s ability to explain graded neuronal responses. Recently, we have shown that pulse-gated synfire chains are capable of propagating graded information coded in mean population current or firing rate amplitudes. In particular, we showed that it is possible to use one synfire chain to provide gating pulses and a second, pulse-gated synfire chain to propagate graded information. We called these circuits synfire-gated synfire chains (SGSCs). Here, we present SGSCs in which graded information can rapidly cascade through a neural circuit, and show a correspondence between this type of transfer and a mean-field model in which gating pulses overlap in time. We show that SGSCs are robust in the presence of variability in population size, pulse timing and synaptic strength. Finally, we demonstrate the computational capabilities of SGSC-based information coding by implementing a self-contained, spike-based, modular neural circuit that is triggered by streaming input, processes the input, then makes a decision based on the processed information and shuts itself down.

## Author Summary

Cognitive tasks are associated with the dynamic excitation of neural assemblies. When we consider how quickly and flexibly such collectives may be formed and incorporated in a task, a persistent question has been: how can the brain rapidly evoke and involve different neural assemblies in a computation, when synaptic coupling changes only slowly? Here, we demonstrate mechanisms whereby information may be rapidly and selectively routed through a neural circuit, and sub-circuits may be turned on and off. The resulting information processing framework achieves the goal that has been pursued, but until now largely not attained, of achieving faithful, flexible information transfer across many synapses and dynamic excitation of neural assemblies with fixed connectivities.

## Introduction

Functioning neuronal networks need to store, transmit, integrate and transform their inputs to achieve the neural computation performed by the brain. How this happens in vivo has not been understood. Many proposed mechanisms rely on rate model formulations with proposed mechanisms ranging from how oscillations are generated [1], to how anatomical architecture can maintain working memory [2], to how long time scales can emerge from the heterogeneous (recurrent, feedforward and feedback) connectivities in the primate cortex [3].

With the advent of modern supercomputing, there have been efforts to simulate the entire (or a large portion of the) brain with realistic spiking neurons [4–6]. There has also been work using spiking models to perform Bayesian inference [7, 8], statistical machine learning algorithms [9], arbitrary, highly accurate linear maps [10], and predictive coding [11].

Numerical studies investigating fundamental computational mechanisms such as information propagation [12–15] have shown that it is possible to transfer firing rates through feed-forward networks when there is sufficient background activity to keep the network near threshold [16]. Further studies have shown that additional coherent spatio-temporal structures (*e.g.* hubs or oscillations) can stabilize the propagation of synchronous activity and select specific pathways for signal transmission [17–20]. Despite many constructive examples, how general computation (i.e. a Turing complete framework) can be performed using spikes or firing rates remains an open problem.

From the above studies and the large literature on this subject, it is seen that there is a range of levels of approximation at which to understand and model neural function. Marr categorized this range [21] as: 1) studies at the *computational* level that ask what the brain does and why, 2) studies at the *algorithmic* level that attack how the brain performs specific computations, the information representations used and the processes that manipulate this information, and 3) studies at the *implementational* level that look for biophysical mechanisms that make up the building blocks to be used at higher levels.

We have demonstrated an information propagation mechanism at the *implementation* level that may be used as a fundamental building block to construct higher level information processing algorithms, and, we hope, may be used to build yet more abstract structures such as the cognitive operating system used by the brain with its many regions and sub-structures [22]. Our mechanism makes use of gating pulses to propagate information in the form of graded pulses from layer to layer. Experimental evidence supports the idea that information in the brain propagates in discrete spike packets, such as the pulses that arise from our mechanism. Luczak, MacNaughton and Harris (LMH) have recently laid out evidence that stereotypical and repeating spike sequences consisting of packets of spikes constitute basic building blocks for neural coding [23]. They show that spike packets have been observed in many different regions of cortex. Thus, they suggest, packet-based information processing is likely to be conserved across much of the brain.

Information packets by themselves are insufficient to form the basis for an information processing system. The system must also be powerful enough to perform general computation. That is, it must be as powerful as a universal Turing machine and be capable of storing, transmitting, integrating and transforming information. In our previous work, we demonstrated that graded information could be faithfully propagated through many layers of a neural circuit and that arbitrary linear maps could be performed using appropriate synaptic connectivities [22]. However, one thing that was lacking was the ability to perform decisions. Once a neural circuit can perform a decision, the circuit itself can control subsequent processing, providing the capacity necessary for general computation. In this paper, after providing a more general understanding of graded information propagation than is discussed in our previous work, we demonstrate that, by allowing graded information to interact with neural gating populations, decisions can be made within our pulse-gated information processing framework. We show how this works by implementing a self-contained, spike-based, modular neural circuit that is triggered by an input stream, reads in and processes the input, generates a conditional output based on the processed information, then shuts itself off.

## Results

**Fig 1:**
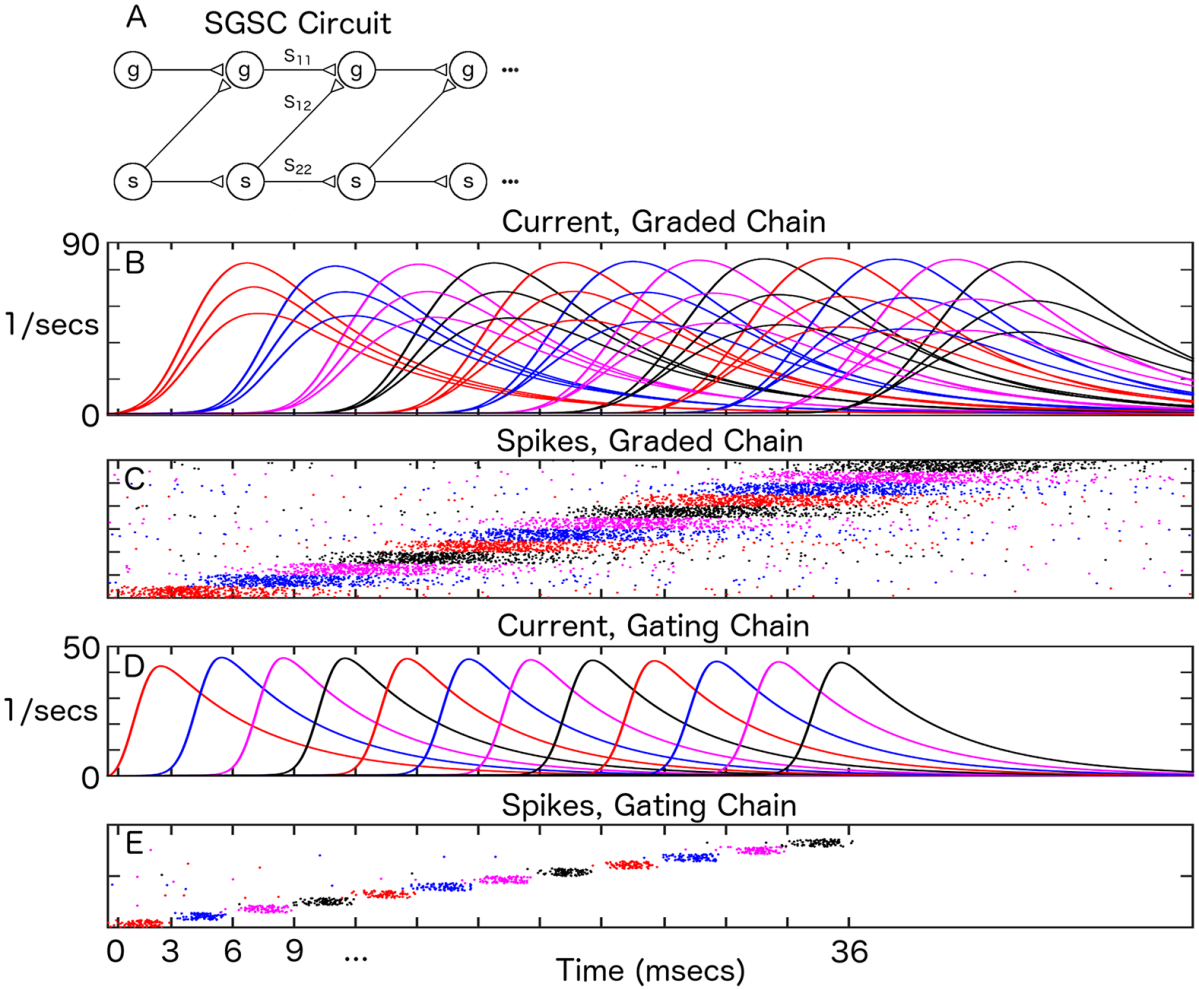
Graded information transfer in synfire-gated synfire chains. A) Circuit diagram. ‘g’ (for ‘graded’) denotes a population in the graded chain. ‘s’ (for ‘synfire chain’) denotes a population in the gating chain. *S*_11_, *S*_12_ and *S*_22_ denote synaptic couplings between and within the respective chains. The gating chain generates pulses that gate the propagation of graded information in the graded chain. B) Mean, synaptic current amplitude transferred across 12 neural populations (labelled red, blue, magenta, black, etc., also see corresponding colors in C) and E)). *N*_1_ = 1000. Averaged over 50 trials. Three amplitudes are depicted. C) Spike rasters from graded populations for one instance of graded transfer. D) Mean, synaptic current amplitude for fixed amplitude synfire chain across 12 layers. *N*_2_ = 100. Averaged over 50 trials. E) Spike rasters from gating populations for one instance of graded transfer. Mean firing rates over the duration of a graded pulse (*T* ∼ 9 ms) reached 30–70 Hz for a single neuron. For the gating chain, the pulse length was shorter (*T* ∼ 3 ms), giving a mean firing rate of 300 Hz for a single neuron. (Please see Materials and Methods for definitions of all parameters.)

In Fig 1, we show how graded information may be propagated in an SGSC neural circuit (Fig 1A). This circuit consists of two feedforward networks. One network (gating chain), set up to operate in the attractor synfire regime, generates a fixed amplitude pulse that propagates from layer to layer (Fig 1D and 1E). The second network (graded chain) receives gating pulses from the gating chain and is capable of propagating graded currents and firing rates from layer to layer (Fig 1B and 1C). The gating chain delivers pulses offset by time *T*_0_ to the graded chain rapidly enough that there is an overlap in the integration of graded information and its transmission from one layer to the next. Graded information, in the form of synaptic currents and firing rates, is faithfully propagated across all 12 layers in the simulation.

**Fig 2:**
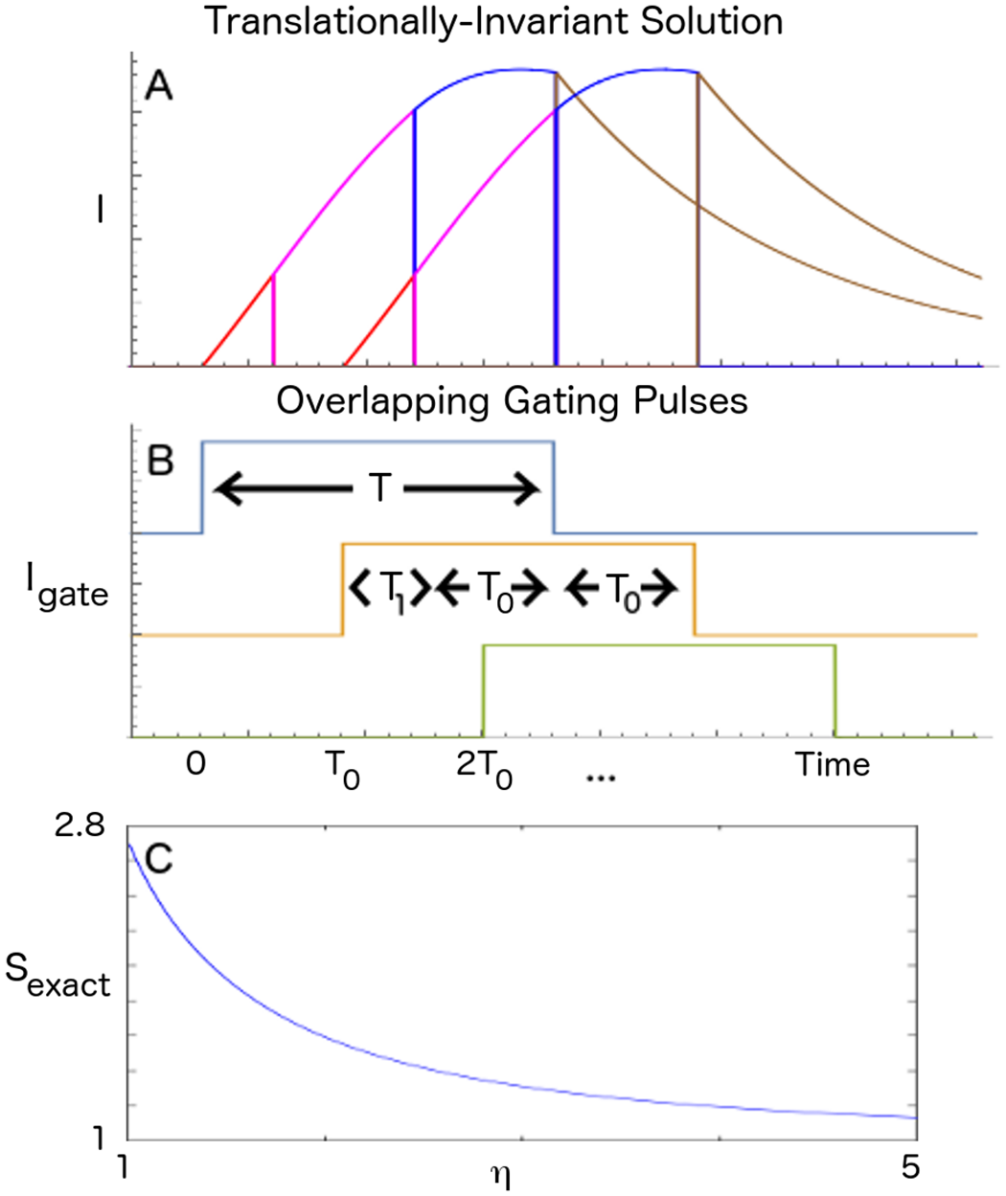
Graded information transfer with overlapping pulses, exact mean-field solution. A) Graded, mean current amplitudes across 2 populations. Two overlapping solutions are shown, one upstream (earlier in time) and one downstream (later in time). The downstream current evolution is easiest to understand: The red segment depicts the epoch when the second gating pulse (depicted in tan) has brought the downstream population to threshold. During this time, the upstream current (depicted in magenta) is integrated and the downstream current begins to rise. Once the upstream current enters the next epoch (depicted in blue), the downstream current (depicted in magenta) continues to rise. After the upstream current begins to decay exponentially (depicted in brown), the downstream current continues to rise (depicted in blue) until the gating pulse ends. At this point, the downstream current decays exponentially. So, from the point of view of the downstream population, the red segment represents the integration of the pink segment of the upstream population, the magenta segment represents the integration of the blue segment of the upstream population, and the blue segment represents the integration of the brown segment of the upstream population. *T*_0_/*τ* = 0.6, *T*_1_/*τ* = 0.3, and *T*/*τ* = 1.5. *S*_*exact*_ for these values is 1.582. The coefficients of the solution polynomial are {0.733, 0.640, 0.228} (See SI Appendix 1). B) Gating pulses offset from 0 for clarity. C) *S*_*exact*_ vs. *η*.

The observation that spike volleys in successive layers of the SGSC overlap in time led us to consider an extension of our previous mean-field model [22] to allow for the integration of graded information in successive populations to overlap in time. As in our previous work, we consider the idealized case in which the gating pulses are square. In Fig 2, we show a translationally invariant solution (Fig 2A) and gating pulses (Fig 2B) from such a mean-field model. Successive gating pulses of length *T* are offset by time *T*_0_. The solution is divided into segments which are the result of the integration of spikes in the corresponding segment (shifted by *T*_0_) from the previous layer during the gating pulse. In the I&F model, both *T* and *T*_0_ result from intrinsic neuronal dynamics and synaptic delays. See Materials and Methods and SI Appendix 2 for the specific parameters that we used in our simulations.

For fixed *T* and *T*_0_, we find time translationally-invariant solutions for synaptic input currents for special values of the feedforward coupling strength, *S* = *S*_*exact*_, in the mean-field model (see Materials and Methods and SI Appendix 1). In Fig 2C, we plot *S*_*exact*_ as a function of *η* = *T*/*T*_0_, where *η* is a measure of the overlap in the integration and transmission of graded information. Note that *S*_*exact*_ becomes flatter as the overlap, *η*, gets larger. This implies that, for large overlaps, any propagation error in the solution due to deviations from *S*_*exact*_ is small. Thus, in the large *η* regime, information propagation is robust to variability in both pulse timing and coupling strength. For practical purposes, we find that *η* > 2 or 3 is sufficiently robust. Furthermore, for a generic feedforward network, there exists a wide range of *S* (roughly, *S* from 1 to 2.7) where we can find time translationally-invariant solutions for which graded propagation is possible.

**Fig 3:**
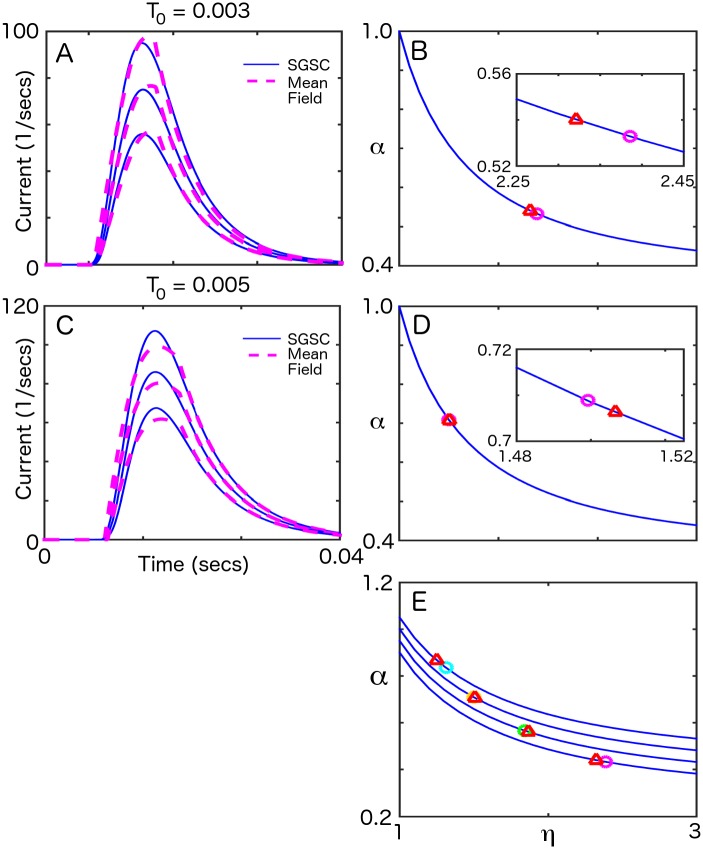
Fitting a square-pulse gated mean-field model of the SGSC. A) Fits of synaptic input currents predicted by the mean-field model to results of I & F simulations for 3 amplitudes for *T*_0_ = 0.003. B) *T*_0_ = 0.003: Blue line—*α*_*exact*_ as a function of *η*. Red triangle—(*η*_*sim*_, *α*_*sim*_), purple circle—(*η*_*fit*_, *α*_*fit*_). Inset: magnification showing location of results from fit. C) Fits of mean-field model and I & F simulation currents for 3 amplitudes for *T*_0_ = 0.005. D) *T*_0_ = 0.005: Blue line—*α*_*exact*_ as a function of *η*. Red triangle—(*η*_*sim*_, *α*_*sim*_), purple circle—(*η*_*fit*_, *α*_*fit*_). Inset: magnification showing location of results from fit. E) Results of fit for *T*_0_ = 0.003, 0.004, 0.005, 0.006. Traces offset for clarity.

In Fig 3, we explore whether our mean-field theory could be used to model our I&F simulation results. First, we determined the parameters (*η*_*fit*_, *α*_*fit*_) that gave the best-fitting mean-field solution to the simulation data, given known *T*_0_. Here, we define *α* ≡ *S*(*T*_0_/*τ*)*e*^−*T*_0_/*τ*^, so that *α* = 1 at *η* = 1. In this expression, *τ* represents a synaptic time scale (see Materials and Methods). Next, using the simulational synaptic coupling, *α*_*sim*_, we found *η* = *η*_*sim*_ that corresponded to the time-translationally invariant solution of the mean-field model. Closeness of these two points would give evidence that the mean-field theory, despite the simplications used to derive it (*e.g.* precisely timed square gating pulses, linear f-I curve, etc.), can be used to model the I&F simulation. We show details of this fitting procedure for two different *T*_0_’s (Fig 3A–3D), and summarize the results for *T*_0_ = 0.003, 0.004, 0.005, 0.006 (Fig 3E). The closeness of model fits with simulation results, for a wide range of overlaps, indicates that the mean-field theory is a good model of the SGSC simulation.

**Fig 4:**
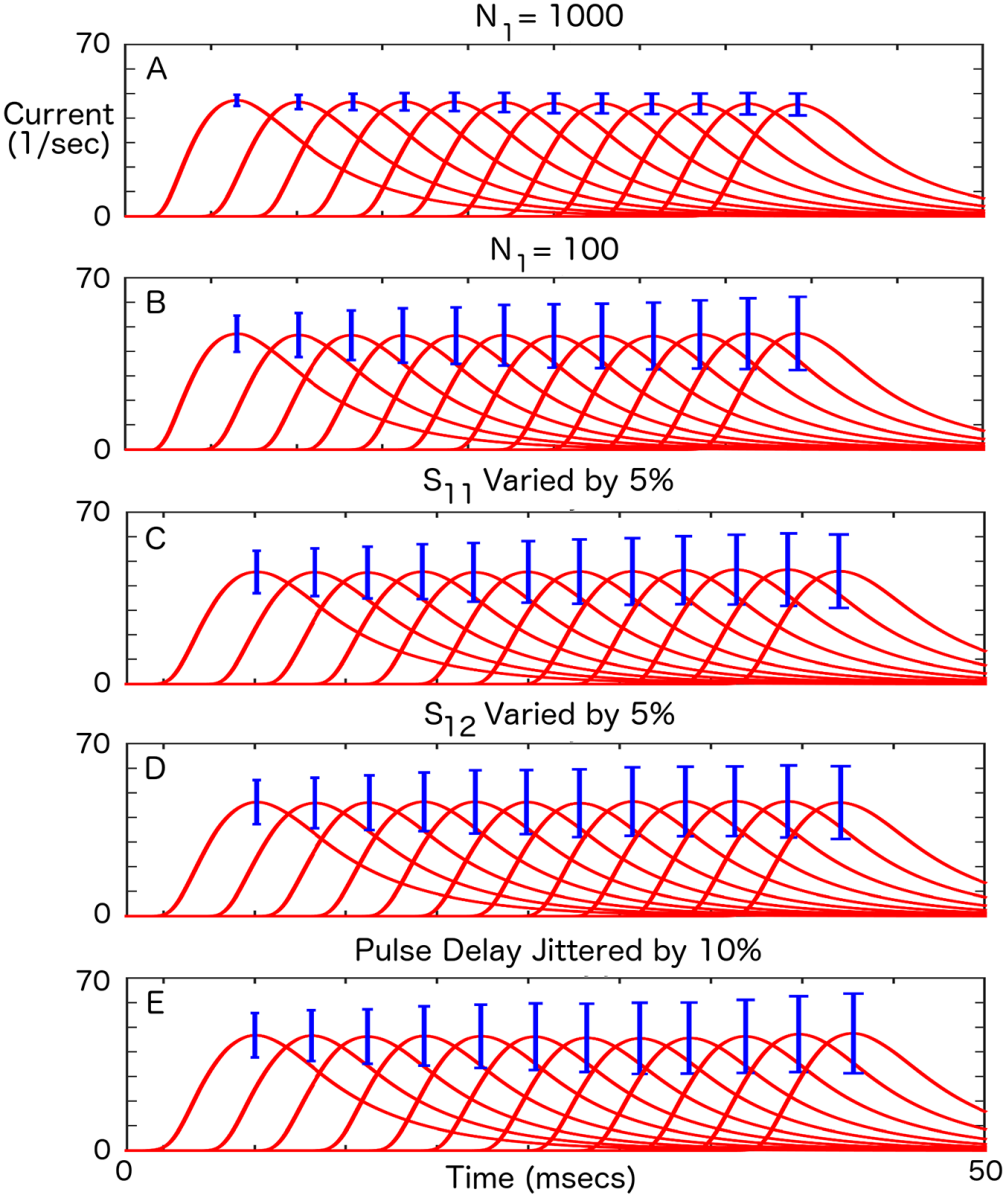
Signal-to-Noise-Ratio as a function of the number of transfers. Red—mean synaptic current amplitude for transfer across 12 layers. Blue—standard deviation of current amplitude. Mean and standard deviation calculated from 1000 trials. A) *N*_1_ = 1000, all subsequent scales are the same. B) *N*_1_ = 100, C) *S*_11_ taken from a uniform distribution with half-width of 5%, *N*_1_ = 100, D) *S*_12_ taken from a uniform distribution with half-width of 5%, *N*_1_ = 100, E) Pulse delay jittered by 10%, *N*_1_ = 100.

In Fig 4, we investigate the robustness of pulse-gated synaptic current transfer in the SGSC to finite-size effects, variability in synaptic coupling, and inaccuracies in pulse timing. As would be expected, transfer variability decreases as $1/\sqrt{N_{1}}$ (Fig 4A and 4B). Randomness in synaptic coupling either in the gating chain or the coupling between chains has little effect on the variability (compare Fig 4C and 4D with Fig 4B). As we mentioned above, this is expected due to the flatness of *S*_*exact*_(*η*) for large *η*. Here, *η* = 2.5. Similarly, jittering *T*_0_ has little effect on the variability of current transfer (Fig 4E).

Pulse-gated propagation mechanisms, such as the SGSC, naturally give rise to a probabilistic, spike-based information processing framework in which information, in the form of spiking probabilities, is processed by graded chains and the flow of information is controlled by gating chains [22]. Additionally, logic operations may be performed by allowing graded information to interact with the pulse generator (see Materials and Methods).

In previous work [22], we demonstrated a number of example circuits that made use of pulse-gated networks to control the flow of graded information through a neural circuit. We showed how a working memory could be constructed. We also showed how matrix transforms, such as a Hadamard transform (a Fourier transform using square-wave, Walsh functions with different frequencies as a basis), could be performed on streaming input. Finally, we showed how pulses could be used to re-entrantly guide information through neural subcircuits to perform iterated computations.

Here, to illustrate the capability of pulse-based information processing to perform complex computations, we show results from a toy model circuit meant to serve a representative function of the brain: to process incoming, time-dependent information, then make a decision based on the processed data. For instance, a similar (non-toy) circuit would implement the recognition of a person’s face, transformed from visual information as it streams into the visual cortex, or the recognition of a word or phrase in the auditory system. Upon recognition, the circuit might send on the information and transfer control to a downstream circuit responsible for the reaction to the recognition.

**Fig 5:**
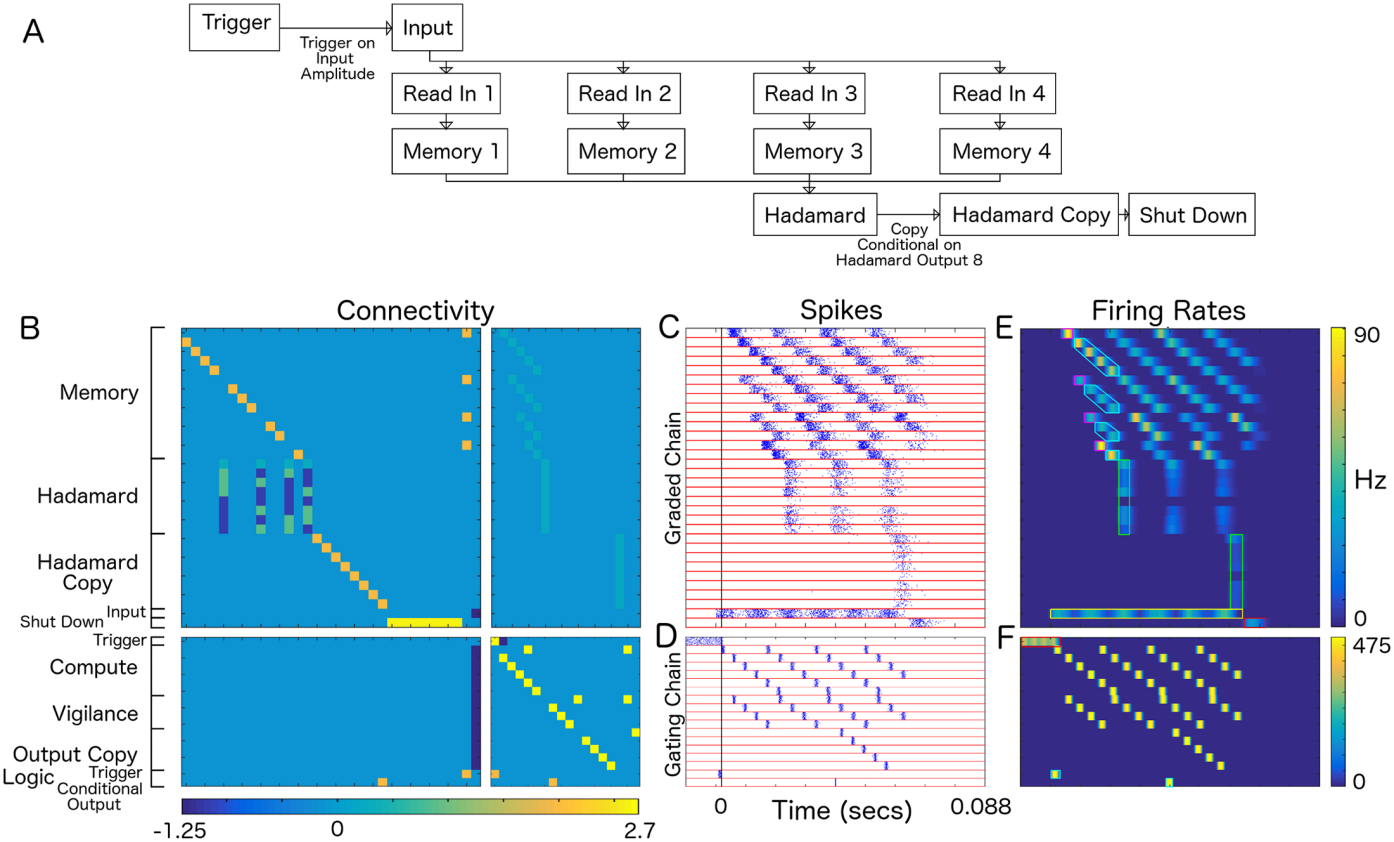
Autonomous decision making circuit. A) Neural Circuit. Input amplitude triggers input to be read in, processed, and, conditionally, the processing output is copied, then the circuit turns itself off. B) Connectivity matrix, divided into four components, *K*^11^ (graded to graded chain connectivities, upper left), *K*^12^ (gating to graded chain connectivities, upper right), *K*^21^ (graded to gating chain connectivities, lower left), and *K*^22^ (gating to gating chain connectivities, lower right). Plotted are values of *K*^*ij*^ times the synaptic connectivity, *S*. Color bar denotes connectivity values. Graded chain populations are: “Memory” (1–14), “Hadamard” (15–22), “Hadamard Copy” (23–30), “Input” (31), and “Shutdown” (32) populations. Memory designates Read In (1, 6, 10, 13) and (non-cyclic) Memory populations. Hadamard designates populations holding Hadamard coefficient amplitudes. The Hadamard transform is divided into two parallel operations, one that results in positive coefficients, the other in absolute values of negative coefficients. Hadamard Copy designates populations into which the Hadamard transform may be copied. Input designates a population that linearly transduces a signal from outside the network. And Shutdown designates a population that receives summed input from the Hadamard Copy populations. Upon excitation, it shuts down the input and gating populations and terminates the computation. Gating chain populations are: “Trigger” (33), a re-entrantly coupled population that fires until inhibited, “Compute” (34–39) for gating the computation of the windowed Hadamard transform in the Memory and Hadamard populations, “Vigilance” (40–43) a pulse loop that, along with the “Logic—Conditional Output” (50) population makes a decision based on the amplitude of the output of the 8’th Hadamard population, and “Output Copy” (44–47) a pulse loop that maintains a memory that the decision was made. Logic populations: “Logic—Trigger” (49) a population that is conditionally excited when both Trigger and Input are excited, and “Conditional Output” (50) a population that is conditionally excited when Hadamard coefficient 8 is above threshold and a population in Output Copy is excited. C) Raster plot showing spikes from the graded chain. *T*_0_ = 4 ms, *T* = 7.5 ms, *τ* = 5 ms. Time runs from left to right. Initially, the Trigger population is re-entrantly excited until the Input amplitude increases. Input, beginning at *t* = −0.025s combines with Trigger to initiate firing in the Logic—Trigger population, which triggers the Compute gating chain and initiates the computation (indicated by vertical black line). Trigger is subsequently turned off by inhibition from the Compute gating chain. We show the computation for three successive windows, each of length 4*T*_0_. The gating chain binds the input into four memory chains of length 4*T*_0_, 3*T*_0_, 2*T*_0_ and *T*_0_. Thus, four temporally sequential inputs are bound in four of the memory populations beginning at times *t* = 4, 8, 12*T*_0_ relative to the trigger. Hadamard transforms are performed beginning at *t* = 5, 9, 13*T*_0_. Each subsequent read in starts one packet length before the Hadamard transform so that the temporal windows are adjacent. At time *t* = 0.06 s, the high amplitude in Hadamard coefficient 8 combines with gating population Conditional Output to initiate the Output Copy chain. The output is copied to Hadamard Copy populations, which then cause the shutdown of the gating chain. D) Raster plot showing spikes from the gating chain. These pulses trigger the computation, gate information through the graded chain, and are also used to perform the decision to copy the Hadamard output to Hadamard Copy for sufficiently high amplitudes in Hadamard, population 8. E) Mean firing rates of the graded chain averaged over 50 realizations. Read In—Magenta, Memory—Cyan, Hadamard Coefficients—Green, Input—Yellow, Shut Down—Red. F) Mean firing rates for the gating chain averaged over 50 realizations. Trigger—Red, Cyan, Output Copy—Cyan. E,F) The firing rates have been smoothed by a moving average process with width 2 ms.

The neural circuit that we demonstrate is triggered by a streaming input. The input is a function that oscillates in time. The circuit encodes the input in graded pulses, then transforms it in order to determine frequency and phase information. It then makes a decision based on the transformed input that affects subsequent processing (Fig 5).

The neural circuit consists of (see Fig 5A) 1) a trigger, 2) a module used to keep sampled streaming input in short-term memory, 3) a 4 × 4 Hadamard transform, 4) a second set of Hadamard outputs (Hadamard Copy) representing output copy to a downstream circuit, 5) an Input population, 6) a Shut Down population to terminate processing, 7) a Compute gating chain to drive the computation, 8) a Vigilance gating chain that serves as a processing indicator and clock to synchronize the triggering of an output decision, 9) an Output Copy gating chain that serves as a decision indicator and is turned on based on the amplitude of the (arbitrarily chosen) 8’th Hadamard coefficient, and 10) Logic populations for a) triggering the computation and b)making the decision to copy the Hadamard output. Output then triggers circuit shutdown by inhibiting all gating chains.

This probabilistic, spike-based algorithm uses a self-exciting population coupled to a streaming input to trigger the computation (see Fig 5B, 5C, 5D and 5E), then continuously gates 4 sequential input amplitudes into 4 read in populations and maintains the input values by gating them through working memory populations until all values are simultaneously in 4 working memory populations. The values in working memory are next gated to Hadamard populations transforming the input values into Hadamard coefficients (one set of positive coefficients and one set of absolute values of negative coefficients [22]). At this point, a time-windowed Hadamard transform has been computed on the input. Input for the simulation in Fig 5 is sinusoidal, so the Hadamard transform, which gives a frequency-based representation of the input, outputs phase and frequency information for the sinusoid. Gating pulses are interleaved such that this computation is performed iteratively on successive windows of length 4*T* from the streaming input.

To implement a conditional copy of the transformed data, we combine the output of the (arbitrarily chosen) eighth Hadamard coefficient in the present Hadamard output (representing that a particular frequency and phase of the input was detected) and the first population in the “Vigilance” gating chain. This operation causes the graded pulse to activate the Output Copy chain when its amplitude is sufficiently high, conditionally causing a pulse to cascade through 4 gating populations with the last population gating the transfer from the subsequent Hadamard output to the 8 output neurons. Once the Hadamard output is copied, it activates the Shutdown population, which inhibits all populations in the gating chains, terminating the computation.

## Discussion

In this Section, we first discuss some technical issues related to the models and circuits that we presented above. Second, we present some ideas for understanding some experimental aspects of spike packets, à la LMH, using our SGSC-based information processing framework. Third, we discuss aspects of oscillatory coherent activity and its possible relationship to pulse-gating. Finally, we discuss some distinctive aspects of information processing using SGSCs and their use.

*Technical Issues:* In parallel circuits such as the decision making circuit depicted in Fig 5, race conditions can exist. This occurs when the relative arrival times of various pieces of information (in our context, graded or gating pulses) are imprecise and thus corrupt a computation. Such a problem could exist in our decision making circuit, however, note that the problem is resolved by the fact that the gating chain (as may be seen in *K*_22_) uses a single chain of populations to gate the working memories and the Hadamard transform. Therefore, as long as gating is consistently timed, there can be no race condition in this circuit, since the pulses that control the parallel transfer of information arise from the same source, and thus gate the entire working memory or Hadamard populations at the same time. Furthermore, due to our use of an attractor synfire chain for gating, the timing is stereotypical and consistent.

The fact that our idealized mean-field model predicts the value and the range of synaptic strengths for exact transfer so well relies on an underlying time-translational invariance of the spiking and membrane potential probability distribution functions. This correspondence can be made more explicit by using a Fokker-Planck approach and suggests that a mean-firing rate description may work well even when the number of neurons in each population is not so large. Our I&F simulations indicate that *N* ≈ 100–1000 is sufficient. Analysis of our Fokker-Planck simulations suggests that the dynamics of graded transfer may be captured by the mean firing rate and a few additional variables encapsulating the equilibrium voltage probability distributions [24]. Ongoing work aims to clarify the conjunctive role of the gating current, the synaptic coupling strength, *S*_*exact*_, and the state of each neuronal population (as described by its membrane potential distribution) in the transmission of firing rates and rate correlations.

*Packets*: In our propagation mechanism, an upstream neuronal population sends spikes downstream, the spikes are integrated in the synaptic current of the downstream population, but unless a gating pulse is provided by a pulse-gating population to the downstream population, the information does not propagate. Gating pulses are provided by a feedforward chain with strong connectivity. Graded information propagates through neural assemblies with sparse, weaker connectivity. LMH conjecture that the cortex rests on a skeleton of stronger connections immersed in a sea of weaker ones. Thus, a correspondence may exist between the skeleton of strong connections and a gating chain and the sea of weakly connected neurons and a graded chain.

Other evidence LMH discuss is: 1) spike-timing reliability progressively decays during a packet giving way to stimulus related firing rates. In our framework, if one considers the combined gating and graded pulses to be a spike packet received by a neuron in a graded population, then the gating pulse occurs at the beginning of a packet, where gating and graded spikes are combined early on. Later spikes in the packet, after the gating pulse ends, consist of spikes from the graded waveform, which decays as spikes are integrated downstream. The early gating pulse would be expected to be the most stereotypical aspect of the mechanism, and gating is purely timing related so many early spikes should be strongly correlated with timing. Also, later spikes (after the gating pulse ends) would consist purely of graded information, and so the transformation to largely firing-rate type statistics would also be expected. 2) Later spiking in a group of packets may represent feedback containing information concerning behavioral choices. We have constructed and demonstrated re-entrant circuits in previous work, that are nonetheless pulse-gated. In these circuits information is retained in packet form, but can be used to modify subsequent circuit properties. So, pulse-gating and feedback are not inconsistent and may be incorporated in the same packet-based information processing system. 3) LMH mention a wide range of functions that packets can serve, including triggering of firing patterns, recall and imagination of sensory stimuli, and attention. The neural circuit of Fig 5 demonstrates three of these functions: pattern triggering via switching on an SGSC, recall in the form of a working memory and attention in the form of the Vigilance gating pulses, and it is straightforward to envision other functions.

*Coherent Activity*: The emerging picture from accumulating experimental evidence is that coherent activity is a fundamental contributor to cognitive function. Accumulating experimental evidence implicates coherent activity as an important element of cognition. Since its discovery [25], activity in the gamma band has been demonstrated to exist in numerous regions of the brain, including hippocampus [26–28], numerous areas in cortex [25, 29–34, 34–37], amygdala and striatum [38]. Gamma band activity is associated with sharpened orientation [39] and contrast [40] tuning in V1, and speed and direction tuning in MT [41]. Attention has been shown to increase gamma synchronization between V4 and FEF [36], LIP and FEF [34], V1 and V4 [42], and MT and LIP [43]. In general, communication between sender and receiver neurons is improved when consistent gamma-phase relationships exist between upstream and downstream sites [30].

Theta-band oscillations are associated with spatial memory [44, 45], where neurons encoding the locations of visual objects and an animal’s own position have been identified [44, 46]. Loss of theta results in spatial memory deficits [47] and pharmacologically enhanced theta improves learning and memory [48].

As defined mathematically (e.g. [49], p. 207), coherence (alternatively, correlation of a signal at a given lag) is a measure of the efficacy of univariate information transfer between neuronal populations. Note that a matrix-valued definition of coherence is needed to measure the efficacy of multivariate information transfer. Here we have demonstrated a coherent transfer mechanism that dynamically routes graded information through a neural circuit using stereotyped gating pulses and makes decisions via non-linear coupling of graded and gating pulses. As we have shown, SGSCs can be used as building blocks to implement complex information processing algorithms, including sub-circuits responsible for short-term memory, linear maps, and computational logic. As such, synfire-gated synfire chains should be considered as a candidate mechanism whenever coherent activity is implicated in information transfer.

We suggested in our previous work that a natural manifestation of neural circuits that repeatedly analyze passive streaming input would be the existence of oscillatory, sub-threshold pulses generated by pulse-gated control signals from gating chains. However, coherent, oscillatory activity in the gamma-band, either spiking activity or sub-threshold voltage oscillations, is typically a transient phenomenon, at least in visual cortex [50]. This makes sense in the computational context that we consider here and is exemplified in the final example in Results. There, processing of streaming information is initiated, requiring repeated (oscillatory) sampling of the input. But the neural circuit is subsequently switched off by logic internal to the circuit. Measurements of such circuits would show transient oscillatory coherent activity. An important implication of pulse-gated information processing for experiment is that the gating rhythms and patterns controlling information flow in a neural circuit will depend on the structure and time scales of the underlying algorithm that the brain implements. For instance, in the circuit in Fig 5, the oscillation frequency is determined by the length of the cyclic gating chains. Thus, different algorithms may be able to be distinguished based on the brain rhythms that they evoke. Alternatively, by observing gating patterns, putative computational algorithms might be able to be determined from brain rhythms.

Rapid visual categorization (RVC) experiments have demonstrated that objects can be recognized as early as 250–300 ms after presentation. It has been conjectured that massively parallel, feedforward networks are used during RVC computations for maximum speed [12, 51–53]. With pulse lengths of 25 ms in an SGSC, 10–12 feedforward processing layers would be needed to construct such a network (Fig 1). The signal-to-noise ratios that we demonstrated for the SGSC (Fig 4) are good enough that it could be used for this type and rate of information transfer. Indeed, in our examples, we show rapid propagation of graded information with 3 ms pulses.

In [54], using calcium imaging in mouse primary visual cortex slices, similar activity to that of a synfire chain was detected. Coactive activity occurred within a 3 ∼ 11ms time window, which is similar to the synfire chain in our SGSC. Spike timing in the spiking pattern was preserved. There is also strong experimental evidence for synfire chains in birdsongs. In [55] and [56], the authors found repeated bursting activity during bird songs that was well-characterized as a synfire chain.

*SGSC*: To our minds, the success of the SGSC graded information propagation mechanism rests on the structural robustness of the pulse gating mechanism. One contribution to robustness is that the synfire chain that is used for gating pulse generation approaches a fixed amplitude attractor with fixed temporal offset. A second contribution is that by providing overlapping temporal windows for information integration, the constraints on parametric precision to achieve graded information transfer are relaxed (Fig 2C and related text). Having said that, the correspondence between our mean-field model and the SGSC gives weight to the idea that pulse-gating, independently of how it is implemented, is a robust mechanism for controlling information transfer in neural circuits. Thus, there is not a particular reason that other pulse generators should not be entertained. For instance, experiments implicate the PVBC/OLM system of interneurons in cortical pulse generation [57].

A conceptual framework for the manipulation of information in neural circuits arises naturally when one considers graded information transfer in the context of coherently interacting neuronal assemblies. In this framework, information processing and information control are conceived of as distinct components of neural circuits [22]. This distinction has been used previously [17, 18, 58, 59] in theoretical mechanisms for gating the propagation of fixed (non-graded) amplitude waveforms. Independently, structures devoted to information gating have been observed experimentally (see [60] for a review). One such circuit is the hippocampus/mediodorsal thalamus (MD)/ventral tegmental area (VTA)/prefrontal cortex (PFC). In this circuit, both MD and VTA have been shown to gate the hippocampal-PFC pathway [61]. Additionally, frontal and basal ganglia activity has been shown to gate access to working memory in human parietal cortex [62]. Here, by providing a mechanism for the propagation of graded information and including computational logic by allowing graded and gating chains to interact, active linear maps (see Materials and Methods) take prominence as a key information processing structure.

It is worth mentioning that when we constructed the neural circuit example in Fig 5, we started at the algorithmic level, then implemented the algorithm in the mean-field firing rate model, then translated the mean-field model into the spiking, I&F network. We feel that this is a major strength of the SGSC-based information processing framework, *i.e.* that it provides a practical pathway for designing computational neural circuits, either for the purpose of forming hypotheses about circuits in the brain, or for implementing algorithms on neuromorphic chips [63].

## Materials and Methods

### The Synfire-Gated Synfire Chain Circuit

Individual current-based, I&F point neurons in the SGSC have membrane potentials described by

$$\begin{array}{c} \frac{d}{dt}v_{i,j}^{\sigma }=-g_{leak}\left(v_{i,j}^{\sigma }-V_{leak}\right)+\sum_{\sigma^{\prime }=1}^{2}I_{i,j}^{\sigma \sigma^{\prime }}+I_{i,j}^{\sigma } \end{array}$$ (1)

$$\begin{array}{c} \tau \frac{d}{dt}I_{i,j}^{\sigma \sigma^{\prime }}=-I_{i,j}^{\sigma \sigma^{\prime }}+\frac{S^{\sigma \sigma^{\prime }}}{p_{\sigma \sigma^{\prime }}N_{\sigma^{\prime }}}\sum_{i^{\prime }}\sum_{l}\delta \left(t-t_{i^{\prime },j-1}^{\sigma^{\prime },l}\right) \end{array}$$ (2)

$$\begin{array}{c} \tau \frac{d}{dt}I_{i,j}^{\sigma }=-I_{i,j}^{\sigma }+f^{\sigma }\sum_{l}\delta \left(t-s_{i,j}^{l}\right) \end{array}$$ (3)

where *σ*, *σ*′ = 1, 2 with 1 for the graded chain and 2 for the gating chain, *i* = 1, …, *N*_*σ*_ denotes the number of neurons per population for each layer, *j* = 1, …, *M* denotes the layer; individual spike times, $\left\{t_{i,j}^{\sigma ,l}\right\}$, with *l* denoting spike number, are determined by the time when $v_{i,j}^{\sigma }$ reaches *V*_*thres*_. The parameters *g*_*leak*_ and *V*_*leak*_ denote the leak conductance and the leak potential. We have used reduced dimensional units in which time retains dimension in seconds and *V*_*thresh*_ − *V*_*leak*_ = 1. In these units *g*_*leak*_ = 50/sec. The parameter *τ* denotes the synaptic timescale (*τ* = 5 ms, or approximately an AMPA synaptic timescale, in the Results above). The current $I_{i,j}^{\sigma \sigma^{\prime }}$ is the synaptic current of the *σ* population produced by spikes of the *σ*′ population. The parameter *S*^*σσ*′^ denotes the synaptic coupling strength and the neurons from layer *σ* to layer *σ*′ are connected in an IID fashion, with coupling probability given by *p*_*σσ*′_. $I_{i,j}^{\sigma }$ is a background noise current generated from Poisson spike times, $\left\{s_{i,j}^{l}\right\}$, with strength *f*^*σ*^ and rate *ν*_*σ*_.

### More Complex Synaptic Processing

General SGSC circuits can incorporate a number of subcircuits, such as short-term memory and processing due to non-trivial synaptic connectivities [22] such as the circuit shown in Fig 5 (Results). In this case, more general connectivities are needed and the above equations become

$$\begin{array}{c} \frac{d}{dt}v_{i,j}^{\sigma }=-g_{leak}\left(v_{i,j}^{\sigma }-V_{leak}\right)+\sum_{\sigma^{\prime }=1}^{2}I_{i,j}^{\sigma \sigma^{\prime }}+I_{i,j}^{\sigma } \end{array}$$ (4)

$$\begin{array}{c} \tau \frac{d}{dt}I_{i,j}^{\sigma \sigma^{\prime }}=-I_{i,j}^{\sigma \sigma^{\prime }}+\frac{S}{p_{\sigma \sigma^{\prime }}N_{\sigma^{\prime }}}\sum_{k}K_{jk}^{\sigma \sigma^{\prime }}\sum_{i^{\prime }}\sum_{l}\delta \left(t-t_{i^{\prime },k}^{\sigma^{\prime },l}\right) \end{array}$$ (5)

$$\begin{array}{c} \tau \frac{d}{dt}I_{i,j}^{\sigma }=-I_{i,j}^{\sigma }+f^{\sigma }\sum_{l}\delta \left(t-s_{i,j}^{l}\right) \end{array}$$ (6)

Here, the synaptic connectivity for the graded chain is $K_{jk}^{11}$, the coupling between the chains is $K_{jk}^{12}$, and the connectivity of the gating chain is $K_{jk}^{22}$. Interaction between the graded chain and the gating chain is given by $K_{jk}^{21}$. We use $K_{jk}^{21}$ to implement conditional logic operations.

### Mean-Field Solutions for Synaptic Current Propagation in the Overlapping Pulse Case

To analyze graded propagation for the case in which the integration of graded information in successive populations overlaps in time, we assume that the gating pulse is square with amplitude sufficient to bring neuronal populations up to the firing threshold. We also assume that above threshold the activity function is linear [22].

Firing in the upstream population is integrated by the downstream population. Thus, the downstream synaptic current obeys

$$\begin{array}{c} \tau \frac{d}{dt}I_{d}=-I_{d}+Sm_{u}\;, \end{array}$$

where *S* is a synaptic coupling strength, *I*_*d*_(*t*) is the downstream synaptic current, and *τ* is a synaptic timescale. In a thresholded-linear model, the upstream firing rate is

$$\begin{array}{c} m_{u}\approx {\left[I_{u}\left(t\right)+I_{0}^{Exc}-g_{0}\right]}^{+}\;, \end{array}$$

where $I_{0}^{Exc}=g_{0}\;p\left(t\right)$ is an excitatory gating pulse, *p*(*t*) = *θ*(*t*) − *θ*(*t* − *T*) and *θ* is the Heaviside step function, causing the downstream population to integrate *I*_*u*_(*t*), giving the current

$$\begin{array}{c} G\left[I_{d}\right]\left(t\right)\equiv Se^{-t/\tau }\left[\int_{0}^{t}ds\;e^{s/\tau }I_{u}\left(s\right)+c\right]\;. \end{array}$$

The graded population is pulsed for time *T*. The offset between successive gating pulses is given by *T*_0_ (see Fig 2, Results). In [22], we studied the case where *T* = *T*_0_. That is, the downstream pulse turned on just when the upstream pulse turned off. Here, we focus on the case where *η* = *T*/*T*_0_ > 1, and *η* need not be an integer. Let *n* be the integer part of *η*. Then *T* = *nT*_0_ + *T*_1_, where *T*_1_ < *T*_0_.

In the SI Appendix 1, we give a general derivation of time translationally invariant solutions in this context. In brief, a translationally invariant, graded current waveform is found for a particular feedforward strength, *S* = *S*_*exact*_, by integrating the upstream firing rate over intervals of length *T*_1_, *T*_0_, …, *T*_0_, while enforcing continuity of the solution. For these solutions, *S*_*exact*_ is given by the smallest root of

$$\begin{array}{c} \sum_{j=0}^{n}\frac{{\left(-1\right)}^{j}}{(n-j)!}{\left[\frac{\left(\left(j+1\right)T_{0}-T_{1}\right)}{\tau }Se^{-T_{0}/\tau }\right]}^{n-j}=0\;. \end{array}$$

### Information Processing Using Graded Transfer Mechanisms

As we demonstrate in Results, current amplitude transfer for the SGSC may be modeled with a piecewise linear activity function, therefore synaptic connectivities between two layers each containing a vector of populations, perform a linear operation on the currents in the upstream layer [22]. For instance, consider an upstream vector of neuronal populations with currents, **I**^*u*^, connected via a connectivity matrix *K* to a downstream vector of neuronal populations, **I**^*d*^:

$$\begin{array}{c} I^{u}\left(t\right)\overset{K}{\to }I^{d}\left(t\right)\;. \end{array}$$ (7)

With feedforward connectivity, *K*, the current amplitude, **I**^*d*^, obeys

$$\begin{array}{c} \tau \frac{d}{dt}I^{d}=-I^{d}+S_{exact}{\left[KI^{u}+p^{u}\left(t\right)-g_{0}\right]}^{+}\;, \end{array}$$ (8)

where **p**^*u*^(*t*) denotes a vector gating pulse on the upstream population. This results in the solution **I**^*d*^(*t* − *T*) = *PK* **I**^*u*^(*t*), where *P* is a diagonal matrix with the gating pulse vector, **p**, of 0s and 1s on the diagonal indicating which neurons were pulsed during the transfer.

This discussion has identified three components of an information processing framework that naturally arises from mechanisms such as the SGSC:

1. information content—graded current, **I**
2. information processing—synaptic weights, *K*
3. information control—pulses, **p**

Note that the pulsing control, **p**, serves as a gating mechanism for routing neural information into (or out of) a processing circuit. We, therefore, refer to amplitude packets, **I**, that are guided through a neural circuit by a set of stereotyped pulses as “bound” information. In the SGSC, information content is carried by the graded chain (*e.g.* Fig 5B and 5D), information processing is performed by the synaptic connectivities (*e.g.* Fig 5A) and information control is performed by the gating chain (*e.g.* Fig 5C and 5E). We will refer to the combination of these control and processing structures as *active linear maps*.

In order to make a decision, non-linear logic circuits are introduced. A simple decision can be implemented in our framework by allowing interaction between information control and content. In our example, a graded and a gating pulse were combined to make a decision, then the output was fed as input to a gating chain. If the graded chain output a low value, the gating chain was not switched on. However, if the graded chain output was high, this initiated pulses in the gating chain, which rapidly approached an attractor. Thus, the interaction caused conditional firing in the gating chain.

## Supporting Information

S1 Appendix Supporting information. (PDF)
