# Supplementary material for: Graded, Dynamically Routable Information Processing with Synfire-Gated Synfire Chains
Source: PLoS Comput Biol. 2016 Jun 16;12(6):e1004979. doi: 10.1371/journal.pcbi.1004979 (PMC4911121; doi:10.1371/journal.pcbi.1004979)
Supplement: S1 Appendix — (PDF) [file pcbi.1004979.s001.pdf]

Graded, Dynamically Routable Information Processing with Synfire-Gated Synfire Chains

Zhuo Wang[1], Andrew T. Sornborger[2,*], Louis Tao[1,3,*],

Supporting Information

Appendix 1: Graded Propagation via Overlapping Gating Pulses

Mean-Field Analysis

To analyze graded propagation for the case in which the integration of graded information in successive populations overlaps, we assume that the gating pulse is square with amplitude sufficient to bring neuronal populations up to the firing threshold. We also assume that above threshold the activity function is linear [**?**].

Firing in the upstream population is integrated by the downstream population. The downstream synaptic current obeys

$$\tau \frac{d}{dt} I_d = -I_d + S m_u \ ,$$

where S is a synaptic coupling strength, $I_d(t)$ is the downstream synaptic current, and τ is a synaptic timescale. For simplicity, we set $\tau = 1$. Therefore, from here on, time will be measured in units of τ. The upstream firing rate is

$$m_u \approx \left[I_u(t) + I_0^{Exc} - g_0 \right]^+ \ ,$$

where $I_0^{Exc} = g_0 \, p(t)$ is an excitatory gating pulse, $p(t) = \theta(t) - \theta(t - T)$ and θ is the Heaviside step function, causing the downstream population to integrate $I_u(t)$, giving the current

$$G\left[I_d\right](t) \equiv S e^{-t} \left[\int_0^t ds \; e^s I_u(s) + c \right] \ .$$

As shown in Fig. 2 (in the main text), we label the offset between upstream and downstream gates by T_0. The solution is broken into a set of $n+1$ equal intervals of length T_0 labelled from later to earlier in time. The $n+1$'st (earliest) interval typically extends before the onset of the pulse, since an arbitrary pulse's length is not an integer multiple of the lag T_0. The delay between the beginning of the final interval and the beginning of the pulse is denoted T_1 (see Fig. 2b). During each interval, the downstream current integrates upstream firing shifted by T_0. The integrated currents and matching conditions for each interval solution are given by

$$\begin{aligned}
G\left[I_0\right](t) &= \quad I_1(t), & I_0(0) &= I_1(T_0) \\
G\left[I_1\right](t) &= \quad I_2(t), & I_1(0) &= I_2(T_0) \\
&\vdots \\
G\left[I_{n-1}\right](t) &= \quad I_n(t), & I_{n-1}(0) &= I_n(T_0) \\
G\left[I_n(s)\theta(s - T_1)\right](t) &= \quad I_{n+1}(t), & I_n(0) &= I_{n+1}(T_0) \\
& & I_{n+1}(T_1) &= 0 \ .
\end{aligned}$$

Here, due to our definition of G, each interval solution begins at time $t = 0$. This leads to the particular form of the matching conditions above. The interval solution will be shifted later on to give a continuous function, made up of the integral solutions, for the synaptic current.

Evaluating, we find

$$\begin{aligned}
I_0(t) &= Se^{-t}c_0 \\
I_1(t) &= Se^{-t}\left[\int_0^t ds\, e^s e^{-s} Sc_0 + c_1\right] \\
&= Se^{-t}\left[Sc_0 t + c_1\right] \\
&\vdots \\
I_j(t) &= Se^{-t}\sum_{i=0}^{j} c_i \frac{(St)^{j-i}}{(j-i)!} \\
&\vdots \\
I_n(t) &= Se^{-t}\sum_{i=0}^{n} c_i \frac{(St)^{n-i}}{(n-i)!} \\
I_{n+1}(t) &= Se^{-t}\left[\int_{T_1}^t ds\, e^s S e^{-s}\sum_{i=0}^{n} c_i \frac{(Ss)^{n-i}}{(n-i)!}\right] \\
&= Se^{-t}\sum_{i=0}^{n} c_i \frac{S^{n-i+1}}{(n-i+1)!}(t^{n-i+1} - T_1^{n-i+1}) \,,
\end{aligned}$$

where $I_0(t)$ is a current due to the exponential decay of the upstream population's firing rate after its integration. Applying the matching conditions, we obtain equations for the coefficients, c_i,

$$I_0(0) = I_1(T_0) \Rightarrow c_0 = e^{-T_0}\left[Sc_0T_0 + c_1\right]$$

and, in general,

$$I_{j-1}(0) = I_j(T_0) \Rightarrow c_{j-1} = e^{-T_0}\sum_{i=0}^{j} c_i \frac{(ST_0)^{j-i}}{(j-i)!} \,.$$

Matching conditions on the final segment

$$\begin{aligned}
I_n(0) &= I_{n+1}(T_0) \\
&\Rightarrow c_n = e^{-T_0}\sum_{i=0}^{n} c_i \frac{S^{n-i+1}}{n-i+1!}(T_0^{n-i+1} - T_1^{n-i+1}) \\
I_{n+1}(T_1) &= 0 \Rightarrow c_{n+1} = 0
\end{aligned}$$

Translating to matrix notation, we define

$$M = \begin{bmatrix}
e^{-T_0}ST_0 - 1 & e^{-T_0} & 0 & \cdots & \cdots & \cdots & 0 \\
e^{-T_0}S^2\frac{T_0^2}{2} & e^{-T_0}ST_0 - 1 & e^{-T_0} & 0 & \cdots & \cdots & 0 \\
e^{-T_0}S^3\frac{T_0^3}{6} & e^{-T_0}S^2\frac{T_0^2}{2} & e^{-T_0}ST_0 - 1 & e^{-T_0} & 0 & \cdots & 0 \\
\vdots & \ddots & \ddots & \ddots & \ddots & \ddots & \vdots \\
e^{-T_0}S^n\frac{T_0^n}{(n)!} & e^{-T_0}S^{n-1}\frac{T_0^{n-1}}{(n-1)!} & \cdots & \cdots & \cdots & \cdots & e^{-T_0} \\
e^{-T_0}S^{n+1}\frac{(T_0^{n+1}-T_1^{n+1})}{(n+1)!} & e^{-T_0}S^n\frac{(T_0^n-T_1^n)}{(n)!} & \cdots & \cdots & \cdots & \cdots & e^{-T_0}S(T_0-T_1) - 1
\end{bmatrix}$$

and

$$\mathbf{c} = [c_0 \; c_1 \; \ldots \; c_n]^T \; .$$

We then solve $\det(M) = 0$ to determine the values, S_i, for which a solution exists. In general, the determinantal equation is an $n+1$'st order polynomial in S. Solution vectors may be found by solving

$$M(S_i)\mathbf{c} = \mathbf{0} \; .$$

For $n < 16$ (as far as we have checked), we find that all $\{S_i\}$ are positive and real and only the smallest eigenvalue, S_{min}, corresponds with a solution vector of all positive coefficients, c_i. For this nonlinear eigenvalue problem, nonlinear Perron-Frobenius theory applies [1, 2]. In particular, for concave self-mappings, T, of the standard cone K into itself, the nonlinear eigenvalue problem has a unique positive eigenvector with positive eigenvalue. There is, therefore, just one graded solution for a given T.

General Expression for Determinant of M

The matrix M is a full Hessenberg matrix with one upper diagonal and all filled lower triangular elements. A general recursive expression for the determinant may be derived in the standard way, i.e. to use row operations (of determinant 1) to reduce M to diagonal form. Then, the determinant is the product of the values, $\{\lambda_i\}$, along the diagonal, $\prod_i \lambda_i$.

We will work with the matrix $M' \equiv e^{-T_0} M$. Note that $\det M = e^{nT_0} \det M'$. With $a_1 = ST_0 - e^{T_0}$, $a_j = \frac{(ST_0)^j}{j!}, j > 1$ and $b_j = \frac{(ST_1)^j}{j!}, j \geq 1$, we have

$$M' = \begin{bmatrix} a_1 & 1 & 0 & \ldots & \ldots & \ldots & 0 \\ a_2 & a_1 & 1 & 0 & \ldots & \ldots & 0 \\ a_3 & a_2 & a_1 & 1 & 0 & \ldots & 0 \\ \vdots & \ddots & \ddots & \ddots & \ddots & \ddots & \vdots \\ a_n & a_{n-1} & \ldots & \ldots & \ldots & a_1 & 1 \\ a_{n+1} - b_{n+1} & a_n - b_n & \ldots & \ldots & \ldots & \ldots & a_1 - b_1 \end{bmatrix}$$

The super diagonal 1s are now eliminated row by row, giving diagonal terms

$$\begin{aligned} \lambda_1 &= a_1 - b_1 \\ \lambda_2 &= a_1 - \frac{a_2 - b_2}{\lambda_1} \\ \lambda_3 &= a_1 - \frac{a_2 - \frac{a_3 - b_3}{\lambda_1}}{\lambda_2} \\ &\vdots \end{aligned}$$

After dividing through by a common denominator, we find

$$\lambda_j = \prod_{m=1}^{j-1} \frac{1}{\lambda_m} \left[\sum_{k=1}^{j} (-1)^{k-1} a_k \prod_{p=1}^{j-k} \lambda_p + (-1)^j b_j \right]$$

This gives a recursion relation for the determinant, $d_n \equiv \det M'_n$, where M'_n denotes the $n \times n$ lower-right hand submatrix of M',

$$d_n \equiv \prod_{m=1}^{n} \lambda_m = \left[\sum_{k=1}^{n} (-1)^{k-1} a_k d_{j-k} + (-1)^n b_n \right]$$

From an investigation of the low order values of this expression, we conjecture that

$$d_n = \sum_{j=0}^{n} \frac{(-1)^j}{(n-j)!} \left[((j+1)T_0 - T_1)\, Se^{-T_0} \right]^{n-j} \ , \tag{1}$$

which we have checked for $n < 16$. This latter expression, (1), is faster to compute than the recursion relation that precedes it.

Appendix 2: Network Parameters for Neural Circuit in Figure 5

Below we provide the network parameters for the simulation presented in Fig. 5.

Population sizes are: $N_1 = 1000$, $N_2 = 100$

Coupling strengths and probabilities are $S^{11} = 2$ with $K^{11}_{jk} = 1$ for memory and Hadamard Copy populations. $K^{11}_{jk} = 0.2$ for the first layer, and $K^{11}_{jk} = \pm 0.5$ for other layers in Hadamard population. $K^{11}_{jk} = 1.3$ for the connectivity from the Hadamard Copy to Shut down population.

The coupling probability $p_{11} = 0.01$.

$S^{22} = 2.7$ with $K^{22}_{jk} = 1$ for all gating populations (Compute, Vigilance and Output Copy). $p_{22} = 0.8$. There is a time delay between layers in the gating population with $t_{delay} = 1$ms. For the Trigger population, we used the same parameters as in the gating populations ($N = 100$, $S = 2.7$, $p = 0.8$, $\nu = 25$Hz, $f = 0.2$, $t_{delay} = 0$, no self-inhibition). The Trigger population is inhibited by the first gating population with $S^{22} = 2.7$, $K^{22}_{jk} = -3.7$. $S^{12} = 0.37$, with $K^{12}_{jk} = 1$ for the connectivity between gating chain and graded chain. $p_{12} = 0.01$.

For the logic populations, $S^{21} = 2$, $K^{21}_{jk} = 1$ and $p_{21} = 1$. The Logic - Trigger population receives inputs from both the Trigger and the Input. The Logic - Conditional Output population receives inputs from the 8th Hadamard population and the first Vigilance population. For the Shutdown population, $S^{21} = 3.4$, $K^{21}_{jk} = -2$, and $p_{21} = 1$. Note that the shut down population inhibits all gating populations and the Input population.

There is self-inhibition for all neurons with $S^{11} = 2$, $K^{11}_{jk} = -0.6$; $S^{22} = 2.7$, $K^{22}_{jk} = -0.5$.

The background Poisson inputs are $\nu^1 = 25$Hz, $f^1 = 0.2$; $\nu^2 = 25$Hz, $f^2 = 0.2$. For all neurons, the refractory period is 2ms.

References

1. Krause U. Perron's stability theorem for non-linear mappings. J Math Econ. 1986;15:275–282.

2. Lemmens B, Nussbaum R, editors. Nonlinear Perron-Frobenius Theory. Cambridge, UK: Cambridge University Press; 2012.
